# Determinants of Statural Growth in European Children With Chronic Kidney Disease: Findings From the Cardiovascular Comorbidity in Children With Chronic Kidney Disease (4C) Study

**DOI:** 10.3389/fped.2019.00278

**Published:** 2019-07-05

**Authors:** Rouven Behnisch, Marietta Kirchner, Ali Anarat, Justine Bacchetta, Rukshana Shroff, Yelda Bilginer, Sevgi Mir, Salim Caliskan, Dusan Paripovic, Jerome Harambat, Francesca Mencarelli, Rainer Büscher, Klaus Arbeiter, Oguz Soylemezoglu, Ariane Zaloszyc, Aleksandra Zurowska, Anette Melk, Uwe Querfeld, Franz Schaefer, and the 4C Study Consortium

**Affiliations:** ^1^Institute of Medical Biometry and Informatics, University of Heidelberg, Heidelberg, Germany; ^2^Department of Pediatric Nephrology, School of Medicine, Cukurova University, Adana, Turkey; ^3^Hôpital Femme Mère Enfant, Hospices Civils de Lyon, Université de Lyon, Lyon, France; ^4^Division of Pediatric Nephrology, Great Ormond Street Hospital, London, United Kingdom; ^5^Department of Pediatrics, Hacettepe University Faculty of Medicine, Ankara, Turkey; ^6^Department of Pediatric Nephrology, Ege University Faculty of Medicine, Izmir, Turkey; ^7^Division of Pediatric Nephrology, Istanbul University Cerrahpasa Faculty of Medicine, Istanbul, Turkey; ^8^Department of Pediatric Nephrology, University Children's Hospital, Belgrade, Serbia; ^9^Pediatric Nephrology Unit, Bordeaux University Hospital, INSERM Unité Mixte de Recherche, Bordeaux, France; ^10^Pediatric Nephrology Unit, Department of Pediatrics, S. Orsola-Malpighi Hospital, University of Bologna, Bologna, Italy; ^11^Pediatric Nephrology, University Children‘s Hospital, University of Duisburg-Essen, Essen, Germany; ^12^Division of Pediatric Nephrology and Gastroenterology, Department of Pediatrics and Adolescent Medicine, Medical University of Vienna, Vienna, Austria; ^13^Department of Pediatric Nephrology, Gazi University School of Medicine, Ankara, Turkey; ^14^CHU Hautepierre, Strasbourg, France; ^15^Department of Pediatric Nephrology, Medical University of Gdansk, Gdansk, Poland; ^16^Department of Pediatric Kidney, Liver and Metabolic Diseases, Hannover Medical School, Hanover, Germany; ^17^Department of Pediatric Nephrology, Charité-Universitätsmedizin Berlin, Berlin, Germany; ^18^Division of Pediatric Nephrology, Center for Pediatrics and Adolescent Medicine, University of Heidelberg, Heidelberg, Germany

**Keywords:** children, chronic kidney disease, height, statural growth, GFR—glomerular filtration rate, anemia, acidosis, hyperparathyroidism

## Abstract

Failure of statural growth is one of the major long-term sequelae of chronic kidney disease (CKD) in children. In recent years effective therapeutic strategies have become available that lead to evidence based practice recommendations. To assess the current growth performance of European children and adolescents with CKD, we analyzed a cohort of 594 patients from 12 European countries who were followed prospectively for up to 6 years in the 4C Study. While all patients were on conservative treatment with a mean estimated glomerular filtration rate of 28 ml/min/1.73 m^2^ at study entry, 130 children commenced dialysis during the observation period. At time of enrolment the mean height standard deviation score (SDS) was −1.57; 36% of patients had a height below the third percentile. The prevalence of growth failure varied between countries from 7 to 44% Whereas patients on conservative treatment showed stable growth, height SDS gradually declined on those on dialysis. Parental height, pubertal status and treatment with recombinant growth hormone (GH) were positively, and the diagnosis of syndromic disease and CKD stage were negatively associated with height SDS during the observation period. Unexpectedly, higher body mass index (BMI) SDS was associated with lower height SDS both at enrolment and during follow up. Renal anemia, metabolic acidosis, and hyperparathyroidism were mostly mild and not predictive of growth rates by multivariable analysis. GH therapy was applied in only 15% of growth retarded patients with large variation between countries. When adjusting for all significant covariates listed above, the country of residence remained a highly significant predictor of overall growth performance. In conclusion, growth failure remains common in European children with CKD, despite improved general management of CKD complications. The widespread underutilization of GH, an approved efficacious therapy for CKD-associated growth failure, deserves further exploration.

## Introduction

Failure of statural growth is one of the major sequelae of chronic kidney disease (CKD) in children (1). Short stature causes psychosocial challenges both during childhood and adult life. Young adults with childhood-onset CKD report a major impact of short body stature on their quality of life (2). Moreover, growth failure is a sensitive indicator of overall morbidity in children with CKD and has been associated with patient mortality (3). However, the interpretation of growth patterns in children with CKD must also take into account genetic and environmental factors e.g., short height and CKD can be shared features of genetically determined syndromes. The physiological growth channel and target height of a patient is determined to a major degree by the height of the parents. On the population level, ethnic and cultural factors as well as macroeconomic conditions may affect growth outcomes.

The etiology of growth failure in CKD is multifactorial. While growth rates are loosely correlated with the overall degree of renal dysfunction (4), the individual degree of growth impairment is believed to reflect variable contributions of individual endocrine and metabolic complications of CKD including accumulation of inhibitors of growth hormone (GH) and insulin-like growth factor (IGF)-1 signaling, the malnutrition-inflammation complex, metabolic acidosis, renal anemia, and hyperparathyroidism (5).

In the past few decades the elucidation of the physiopathological basis of CKD-associated growth failure and the advent of effective growth promoting therapies have allowed improved clinical management. A moderate improvement of final adult height over time has been documented in children with end-stage kidney disease (6). Notably, improved growth appears to be limited to the period before renal replacement therapy is instituted, whereas growth patterns on dialysis and post-transplantation have shown little change with time.

In the past decade, the 4C Study has followed a large unselected European cohort of children with advanced CKD (7, 8). This study provides an excellent opportunity to analyze the growth outcomes achieved with contemporaneous CKD management and to identify any major clinical and biochemical factors that continue to be associated with suboptimal statural growth in children with CKD.

## Materials and Methods

### Study Population

A total of 704 children aged 6–17 years with eGFR between 10 and 60 mL/min/1.73 m^2^ were enrolled for the 4C Study between January 2010 and May 2012 at 54 pediatric nephrology units in 12 European countries and followed by 6-monthly study visits until December 2018. Only children with active systemic vasculitis, renal vascular anomalies, or primary cardiovascular anomalies preventing assessment of cardiovascular status were excluded from the study. The detailed study protocol has been published previously (7). The study was approved by the Ethics Committee of the University of Heidelberg (S-032/2009) and the institutional review boards at each participating institution. Written informed consent was obtained from all parents and participants as appropriate.

In the analysis presented here, all patients with at least one valid baseline and follow-up height measurement were included. All follow-up visits were included during a 6 years observation period unless patients reached age 18 (male) or 16 years (female) or received preemptive kidney transplantation. According to these criteria, 594 patients with a total of 4,036 visits were included in the analysis. The subgroup with information on target height comprised 484 patients with 3,405 visits.

### Definition of Variables

eGFR was calculated using the Schwartz bedside formula [eGFR = 0.413 × height (cm)/serum creatinine (mg/dl)] (9). Body mass index (BMI) was normalized by calculation of standard deviation scores (SDS) based on reference values of healthy children (10). For calculation of height SDS, synthetic reference data sets published for Northern Southern European populations were used (11). Target height (TH) was calculated based on the height of father (FH) and mother (MH) as *TH*_*boys*_ = 44.5+0.376·*FH*+0.411·*MH*, *TH*_*girls*_ = 47.1+0.334·*FH*+0.364·*MH*, or based on maternal height if father's height was not available: *TH*_*boys*_ = 99.9+0.492·*MH*, *TH*_*girls*_ = 96.3+0.436·*MH* (12).

### Statistical Analysis

Data are given as mean (SD), median (interquartile range), or frequencies (*n*, %). Baseline characteristics are presented for the whole sample, stratified by CKD stage, and for the subgroup with information on target height. Associations at baseline were analyzed using multivariable linear models. These associations were analyzed in the whole sample and in the subgroup of patients with information on target height, where target height SDS was included as covariate in the model.

Longitudinal analyses were performed using multivariable linear mixed effects models with random intercept and slope per patient to account for patient-individual deviations from the population mean trajectories. PTH and CRP were log-transformed to account for the skewed distribution of these parameters. Three models were considered with different covariates included (nested models) and Akaike Information Criterion (AIC) is given for comparison of model fit.

To assess whether the association of a time-dependent covariate and the respective outcome was stable over time, interaction effects with time since study entry were added to the models. The trajectory of height SDS was calculated using a generalized additive mixed effects model, accounting for patient dropout with a penalized spline fixed effect for time since study entry and patient-individual random intercept and slopes. Missing covariate data was imputed five times by the MICE package in R using 2l.norm function (13) and all variables as predictors to minimize bias. Model-based inference was conducted using Rubin's Rule to combine the results obtained from the different imputed data sets (14). AIC is given as the mean AIC of the five imputed models. *P* < 0.05 was considered statistically significant but not adjusted for multiple testing due to the exploratory nature of this study. Data were analyzed using SAS version 9.4 and R version 3.5.1.

## Results

### Study Population

The patient characteristics at time of enrolment are given in Table 1. Two thirds of the patients were male, 39% pubertal, 71% had CAKUT as underlying renal diagnosis and in 6.8% a defined syndrome had been diagnosed. The syndromic disorders encompassed ciliopathies [Bardet-Biedl (*n* = 7), Senior Loken (*n* = 1), Joubert (*n* = 1)], chromosomal aberrations (*n* = 3), branchio-oto-renal syndrome (*n* = 3), prune belly (*n* = 2), Hirschsprung disease with renal hypoplasia (*n* = 2), and a wide range of single cases of mostly monogenic disorders.

**Table 1:** Baseline characteristics for the total cohort, the subgroup with information on target height, and the total cohort stratified by CKD stage.

	**All**	**Subgroup with target height info**	**CKD stage 2**	**CKD stage 3a**	**CKD stage 3b**	**CKD stage 4**	**CKD stage 5**
*N*	594	484	13	42	164	284	74
Age (years)	11.7 (3.2)	11.7 (3.2)	12.4 (2.6)	11.9 (3.1)	11.6 (2.9)	11.5 (3.3)	12.2 (3.4)
Male	395 (68.5%)	333 (68.8%)	6 (46.2%)	28 (66.7%)	114 (69.5%)	197 (69.4%)	50 (67.6%)
Pubertal	222 (38.5%)	182 (37.6%)	6 (46.2%)	17 (40.5%)	58 (35.4%)	109 (38.4%)	32 (43.2%)
Diagnosis							
CAKUT	410 (71.1%)	335 (69.2%)	5 (38.5%)	33 (78.6%)	115 (70.1%)	207 (72.9%)	50 (67.6%)
Tubulointerstitial disorders	75 (13.0%)	70 (14.5%)	4 (30.8%)	1 (2.4%)	21 (12.8%)	40 (14.1%)	9 (12.2%)
Glomerulopathies	44 (7.6%)	38 (7.9%)	2 (15.4%)	4 (9.5%)	9 (5.5%)	21 (7.4%)	8 (10.8%)
Post-AKI CKD	27 (4.7%)	25 (5.2%)	1 (7.7%)	2 (4.8%)	12 (7.3%)	10 (3.5%)	2 (2.7%)
Other	21 (3.6%)	16 (3.3%)	1 (7.7%)	2 (4.8%)	7 (4.3%)	6 (2.1%)	5 (6.8%)
Defined syndrome	35 (6.7%)	31 (6.9%)	0 (0.0%)	3 (8.3%)	8 (5.6%)	22 (8.3%)	2 (3.1%)
Country of origin							
Turkey	277 (48.0%)	231 (47.7%)	11 (84.6%)	18 (42.9%)	73 (44.5%)	139 (48.9%)	36 (48.6%)
Germany	87 (15.1%)	75 (15.5%)	1 (7.7%)	4 (9.5%)	24 (14.6%)	44 (15.5%)	14 (18.9%)
France	51 (8.8%)	42 (8.7%)	0 (0.0%)	6 (14.3%)	21 (12.8%)	23 (8.1%)	1 (1.4%)
Italy	45 (7.8%)	32 (6.6%)	0 (0.0%)	4 (9.5%)	13 (7.9%)	19 (6.7%)	9 (12.2%)
UK	31 (5.4%)	28 (5.8%)	0 (0.0%)	1 (2.4%)	6 (3.7%)	19 (6.7%)	5 (6.8%)
Poland	27 (4.7%)	18 (3.7%)	0 (0.0%)	2 (4.8%)	14 (8.5%)	7 (2.5%)	4 (5.4%)
Austria	17 (2.9%)	17 (3.5%)	0 (0.0%)	2 (4.8%)	1 (0.6%)	12 (4.2%)	2 (2.7%)
Serbia	16 (2.8%)	16 (3.3%)	0 (0.0%)	4 (9.5%)	5 (3.0%)	5 (1.8%)	2 (2.7%)
Other	26 (4.5%)	25 (5.2%)	1 (7.7%)	1 (2.4%)	7 (4.3%)	16 (5.6%)	1 (1.4%)
Height (cm)	139.5 (20.2)	138.8 (20.3)	142.9 (16.5)	143.5 (20.6)	140.8 (19.0)	137.4 (20.8)	139.3 (20.2)
Height (SDS)	−1.52 (1.47)	−1.61 (1.48)	−1.40 (1.40)	−1.12 (1.32)	−1.21 (1.26)	−1.68 (1.47)	−1.94 (1.61)
Height <3rd pct	205 (35.5%)	180 (37.2%)	4 (30.8%)	9 (21.4%)	44 (26.8%)	112 (39.4%)	36 (48.6%)
Target height (cm)		173.3 (7.6)	168.3 (7.4)	173.1 (7.5)	174.1 (7.6)	173.3 (7.6)	173.1 (7.6)
Target height (SDS)		−0.09 (0.70)	−0.37 (0.61)	−0.06 (0.63)	−0.03 (0.69)	−0.10 (0.74)	−0.12 (0.66)
Target height deficit (SDS)		−1.52 (1.47)	−1.08 (1.52)	−1.13 (1.22)	−1.26 (1.24)	−1.62 (1.46)	−1.82 (1.70)
BMI (kg/m^2^)	18.2 (3.8)	18.2 (3.9)	17.4 (1.7)	18.5 (3.4)	18.1 (4.1)	18.1 (3.9)	18.1 (3.2)
BMI (SDS)	0.08 (1.32)	0.08 (1.32)	−0.04 (1.04)	0.15 (1.14)	−0.07 (1.39)	0.10 (1.32)	0.19 (1.12)
BMI <5th pct	50 (8.7%)	42 (8.6%)	0 (0.0%)	3 (7.1%)	16 (9.8%)	27 (9.5%)	4 (5.4%)
BMI > 85th pct	127 (22.0%)	109 (22.5%)	2 (15.4%)	10 (23.8%)	38 (23.2%)	60 (21.1%)	17 (23.0%)
GH therapy	53 (9.2%)	44 (9.1%)	1 (7.7%)	2 (4.8%)	13 (7.9%)	25 (8.8%)	12 (16.2%)
Steroid therapy	13 (2.2%)	11 (2.3%)	0 (0.0%)	0 (0.0%)	4 (2.4%)	6 (2.1%)	2 (2.7%)
eGFR (ml/min/1.73 m^2^)	28.4 (13.5)	27.3 (12.9)	77.3 (16.6)	51.3 (4.1)	36.2 (4.0)	22.4 (4.5)	12.1 (2.1)
Hemoglobin (g/dl)	11.7 (1.6)	11.7 (1.6)	12.5 (1.6)	12.5 (1.3)	12.0 (1.5)	11.5 (1.5)	11.2 (1.8)
Serum bicarbonate (mM)	21.3 (3.7)	21.3 (3.7)	24.8 (4.7)	22.5 (2.8)	21.4 (3.4)	21.3 (3.7)	20.2 (4.1)
Serum phosphate (mM)	1.55 (0.35)	1.55 (0.35)	1.57 (0.75)	1.49 (0.38)	1.47 (0.33)	1.55 (0.28)	1.72 (0.45)
Serum albumin (g/L)	39.0 (5.5)	39.11 (5.40)	39.5 (8.5)	39.7 (3.8)	38.6 (5.3)	39.2 (5.6)	38.7 (5.9)
PTH^*^ (uM)	12.8 (16.1)	13.57 (16.2)	5.9 (7.1)	7.5 (7.1)	10.3 (9.6)	15.3 (16.9)	18.4 (22.1)
CRP^*^ (mg/dl)	0.55 (1.83)	0.57 (1.95)	0.38 (0.83)	0.58 (1.57)	0.51 (1.90)	0.55 (1.80)	0.63 (2.89)

Data are N (%), mean (SD), or (marked with ^*^) median (interquartile range).

Patients from Turkey comprised almost half of the cohort. During the observation period, 130 children were followed on dialysis (61 hemo-, 69 peritoneal dialysis) for a mean (SD) time of 3.6 (2.3) years. Two hundred (34%) patients were censored when they reached the end of the growth period (age 18 years in boys, 16 years in girls), 156 (26%) because of pre-emptive transplantation, and 204 (34%) due to dropout mainly related to loss from follow-up.

At study entry hemoglobin and serum bicarbonate levels slightly decreased and serum phosphate, PTH and CRP levels increased by CKD stage. The prevalence of short stature increased from 21% at CKD stage 3a to 46% at CKD stage 5 and the fraction of patients receiving recombinant growth hormone (GH) therapy rose from 5 to 16%. During follow-up, 92 patients received growth hormone for an average of 72.7 (range 2.7–100) % of the observation time. The BMI SDS was close to 0 at all CKD stages, indicating the absence of major nutrition abnormalities. Oral steroid therapy was administered at any time during the observation period in 20 patients (thereof 13 with glomerulopathies) at a median (IQR) daily prednisone equivalent dose of 0.14 (0.63) mg/kg. The median (IQR) treatment time including pre-enrolment periods was 17.3 (32.8) months.

Table 2 shows key patient characteristics according to country of residence. Short stature was most prevalent in Turkey (45%) and the UK (39%) and least common in Germany (7%). Malnutrition was most common in Serbia (19%) whereas the highest prevalence of overweight and obesity was observed in the UK (42%). Whereas, pharmacological treatment of renal anemia, metabolic acidosis, and hyperparathyroidism did not show major variation between countries, GH use ranged from <1 to 50% of patients.

**Table 2:** Key patient characteristics at study entry and chronic medications administered during observation period according to country of residence.

	**Turkey**	**Germany**	**France**	**Italy**	**UK**	**Poland**	**Austria**	**Serbia**	Other^*^
*N*	284	87	51	46	31	35	17	16	27
Age (y)	11.7 (3.1)	11.7 (3.2)	11.3 (3.3)	11.5 (3.4)	11.3 (3.1)	12.7 (2.9)	13.3 (3.6)	11.0 (3.2)	11.3 (3.4)
% CAKUT/glomerulopathy/other	74/6/20	62/14/24	63/2/35	72/11/17	74/19/7	80/0/20	76/12/12	94/0/6	63/7/30
% syndromic disease	10.9	12.6	21.6	15.2	12.9	14.3	5.9	6.3	7.4
eGFR (ml/min/1.73m^2^)	28.9 (15.3)	26.5 (11.8)	31.9 (10.8)	26.3 (12.0)	23.8 (9.3)	30.1 (10.4)	24.7 (11.4)	31.8 (13.5)	29.3 (12.8)
Height SDS	−1.96 (1.63)	−0.69 (1.15)	−1.30 (0.97)	−1.46 (1.06)	−1.53 (1.17)	−0.85 (1.20)	−1.10 (1.01)	−1.58 (1.10)	−1.15 (1.50)
% height < −2 SDS	45.1	6.9	23.5	23.9	38.7	14.3	11.8	31.3	25.9
BMI SDS	0.10 (1.38)	0.07 (1.09)	−0.10 (1.29)	−0.08 (1.49)	0.60 (1.29)	0.07 (1.19)	0.07 (1.05)	−0.26 (1.88)	0.04 (1.19)
% malnourished	9.2	5.7	11.8	10.9	3.2	2.9	5.9	18.8	11.1
% overweight/ obese	23.6	19.5	15.7	23.9	41.9	22.9	11.8	25.0	14.8
% patients receiving medication during observation period									
ESA	41.2	65.5	54.9	52.2	83.9	31.4	52.9	50.0	55.6
Active vitamin D	69.7	89.7	86.3	89.1	100.0	88.6	94.1	87.5	88.9
Bicarbonate	72.9	81.6	80.4	84.8	67.7	51.4	94.1	75.0	81.5
Recombinant GH	0.7	43.7	39.2	28.3	6.5	8.6	11.8	50.0	18.5
Steroids	4.6	2.3	2.0	4.4	0.0	0.0	5.9	0.0	3.7

*ESA, erythropoiesis stimulating agents; GH, human growth hormone*.

* *Other': Switzerland (n = 11), Lithuania (n = 6), Portugal (n = 6), Czech Republic (n = 4)*.

### Factors Associated With Height at Study Entry

Standardized height at time of enrolment was strongly positively associated with the genetic target height (0.5 height SD per 1 SD unit), being pubertal (0.75 height SD), and the level of preserved GFR (0.18 height SDS per 10 ml/min/1.73 m^2^) (Table 3). While the type of underlying kidney disease did not impact on height, patients with syndromic disorders were 0.3 SD shorter than those with isolated kidney disease. Modest inverse associations were also observed with age (−0.3 height SD per 5 years) and BMI (−0.13 height SD per 1 BMI SD unit). In addition to these biological factors, the country of residence was an important independent predictor of height SDS at study entry. Patients in Turkey, Serbia, Italy, France, and the UK were significantly shorter than patients in Germany. These associations were consistent in the subgroup with available target height information (Table 3) and the total cohort (Table S-1).

**Table 3:** Multivariable linear model of factors associated with height SDS at time of study enrolment in subgroup with target height information.

	**Estimate**	**95% confidence interval**	***P*-value**
Intercept	−0.87	−1.57; −0.18	0.014
Age (years)	−0.04	−0.10; 0.02	0.144
Female sex	−0.17	−0.44; 0.09	0.197
Target height SDS	0.50	0.33; 0.67	<0.001
Diagnosis			
CAKUT (reference)	0.00	–	–
Glomerulopathies	0.28	−0.19; 0.75	0.238
Tubulointerstitial disorders	0.12	−0.22; 0.46	0.485
Post-AKI CKD	−0.06	−0.62: 0.51	0.841
Other	0.43	-0.25; 1.10	0.213
Syndromic disease	−0.30	−0.68; 0.08	0.117
Status: pubertal	0.69	0.32; 1.06	<0.001
BMI SDS	−0.09	−0.18; 0.00	0.052
eGFR (per 10 ml/min/1.73 m^2^)	0.18	0.08; 0.27	<0.001
Previous time on GH (years)	0.05	-0.05; 0.15	0.316
Cumulative prednisone dose (mg/kg)	0.00	-0.00; 0.00	0.897
Country of residence			
Germany (reference)	0.00	–	–
Turkey	−1.37	−1.73; −1.01	<0.001
Serbia	−1.08	−1.79; −0.36	0.003
Italy	−0.83	−1.38; −0.28	0.003
France	−0.74	−1.24; −0.24	0.004
UK	−0.65	−1.23; −0.07	0.027
Austria	−0.44	−1.13; 0.26	0.221
Poland	−0.28	−0.96; −0.40	0.424
Other countries^*^	−0.38	−0.98; 0.22	0.214

* *Czech Republic, Lithuania, Portugal, Switzerland*.

### Factors Associated With Longitudinal Growth During Observation Period

**Figure 1:**
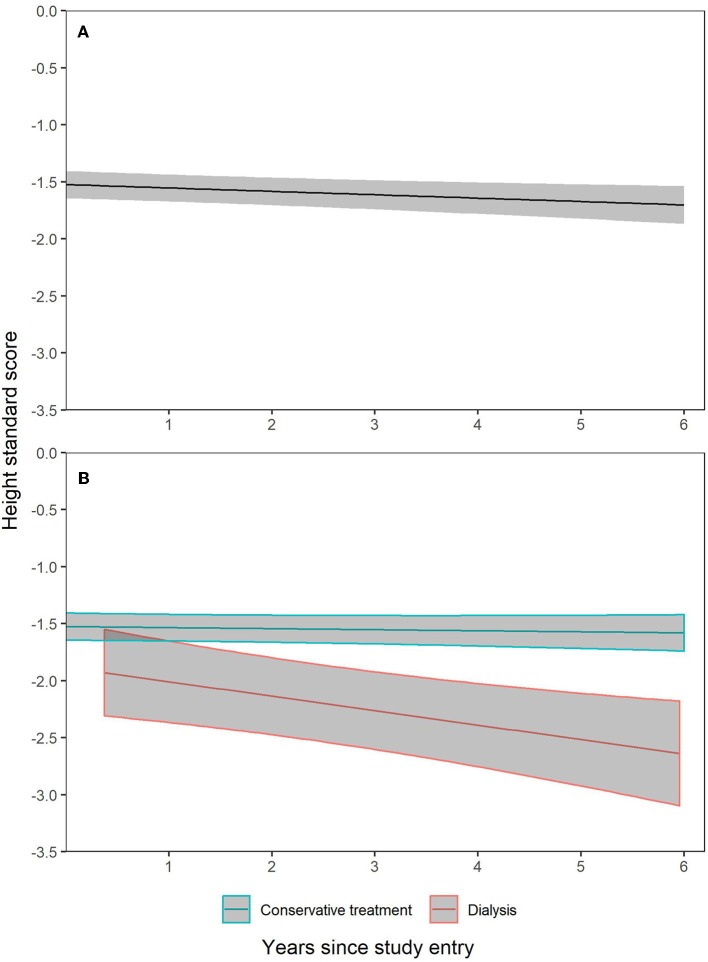
Height SDS trajectories with 95% confidence intervals during 6 years prospective follow-up. Patient dropout was accounted for by construction of generalized additive mixed effects model with penalized spline fixed effect for time since study entry and patient individual random intercept and slopes. **(A)** is based on all observations in all patients. In **(B)**, separate trajectories were constructed for CKD patients followed on conservative treatment and those who switched to dialysis.

The trajectory of height SDS during the 6 years observation period is shown for the total cohort in Figure 1A. It demonstrates minimal loss of standardized height over time. Three multivariable models were calculated to identify factors associated with the course of height SDS during prospective observation (Table 4). The first model considers the impact of renal function according to CKD stage; in the second model GFR-dependent biochemical parameters with potential impact on growth were added; and in the third model the country of residence was included. The renal diagnosis categories were not associated with the course of height SDS and therefore omitted from the models.

**Table 4:** Factors associated with height SDS during prospective observation period.

		**Model 1 (AIC = 4781.2)**			**Model 2 (AIC = 4841.0)**			**Model 3 (AIC = 4809.7)**	
	**Estimate**	**95% CI**	***P*-value**	**Estimate**	**95% CI**	***P*-value**	**Estimate**	**95% CI**	***P*-value**
Intercept	−1.54	−2.01; −1.07	<0.001	−1.47	−1.98; −0.97	<0.001	−0.73	−1.28; −0.18	0.010
Time (years)	−0.06	−0.08; −0.03	<0.001	−0.06	−0.08; −0.03	<0.001	−0.05	−0.08; −0.03	<0.001
Age at enrolment (years)	0.01	−0.03; 0.04	0.799	0.01	−0.03; 0.04	0.792	0.00	−0.03; 0.04	0.877
Female sex	−0.24	−0.49; 0.01	0.06	−0.24	−0.49; 0.01	0.061	−0.13	−0.37; 0.12	0.307
Syndromic disease	−0.37	−0.72; −0.01	0.042	−0.36	−0.71; −0.01	0.045	−0.38	−0.72; −0.04	0.027
Status: pubertal	0.16	0.12; 0.20	<0.001	0.16	0.12; 0.20	<0.001	0.16	0.12; 0.20	<0.001
BMI SDS	−0.14	−0.16; −0.13	<0.001	−0.14	−0.16; −0.13	<0.001	−0.14	−0.16; −0.13	<0.001
Cum. hospitalization time (mo)	−0.08	−0.17; 0.01	0.074	−0.08	−0.17; 0.01	0.077	−0.08	−0.17; 0.01	0.068
Cum. time on GH (years)	0.19	0.14; 0.23	<0.001	0.19	0.14; 0.23	<0.001	0.17	0.12; 0.21	<0.001
Cum. prednisone dose (mg/kg)	−0.00	-0.00; 0.00	0.227	−0.00	−0.00; 0.00	0.237	−0.00	−0.00; 0.00	0.287
CKD stage									
2	0.10	−0.03; 0.22	0.129	0.10	−0.03; 0.23	0.128	0.10	−0.02; 0.23	0.110
3a	0.04	−0.03; 0.11	0.269	0.04	−0.03; 0.11	0.295	0.04	−0.03; 0.11	0.255
3b (reference)	0.00	-	–	0.00	–	–	0.00	–	–
4	−0.00	−0.06; 0.05	0.902	−0.00	−0.06; 0.05	0.958	−0.00	−0.06; 0.05	0.920
5	−0.06	−0.13; 0.00	0.052	−0.06	−0.12; 0.01	0.083	−0.06	−0.12; 0.00	0.075
Dialysis	−0.13	−0.22; −0.05	0.002	−0.12	−0.21; −0.04	0.005	−0.12	−0.21; −0.03	0.006
Hemoglobin (g/dl)				−0.00	−0.01; 0.01	0.461	−0.00	−0.01; 0.01	0.421
Serum albumin (g/L)				0.00	−0.00; 0.00	0.945	0.00	−0.00; 0.00	0.991
Serum bicarbonate (mM)				−0.00	−0.01; 0.00	0.530	−0.00	−0.01; 0.00	0.437
Serum phosphorus (mM)				−0.02	−0.05; 0.01	0.153	−0.02	−0.05; 0.01	0.163
log [PTH (uM)]				0.01	-0.01; 0.02	0.227	0.01	−0.01; 0.02	0.191
log [CRP (mg/L)]				−0.01	−0.01; 0.00	0.165	−0.01	−0.01; 0.00	0.202
Country of residence									
Germany (reference)							0.00	–	–
Turkey							−1.12	−1.46; −0.79	<0.001
Serbia							−0.87	−1.61; −0.14	0.020
Italy							−0.76	−1.25; −0.27	0.002
France							−0.58	−1.06; −0.11	0.017
UK							−0.60	−1.16; −0.03	0.038
Austria							−0.39	−1.11; 0.33	0.286
Poland							−0.08	−0.62; 0.47	0.781
Other countries							−0.47	−1.06; 0.13	0.125

*Effects resulting from models without time interactions are shown. CI, confidence interval; AIC, Akaike Information Criterion*.

The analysis demonstrated positive effects of entering puberty and cumulative time of GH treatment on height SDS, and a negative impact of CKD stage 5 and dialysis, syndromic disease, a higher BMI SDS and, at borderline significance, cumulative hospitalization time. The difference in growth pattern of patients in whom dialysis was initiated vs. those in earlier stages of CKD on continued conservative treatment is illustrated in Figure 1B.

The association of CKD5 and dialysis with growth failure was not explained by variation of hemoglobin, serum bicarbonate, PTH, phosphorus or CRP (model 2). The country of residence was highly predictive of the height SDS even when accounting for all above factors (model 3).

To assess whether the effect of the variables associated with height SDS in the longitudinal model changed with time of observation, interaction terms were added to model 3 (Table S-2). This analysis disclosed that the negative impact of dialysis on height SDS increased with time (estimate −0.05; 95% CI −0.09 to −0.01; *p* = 0.015). Likewise, the inverse association of BMI SDS and height SDS became stronger with observation time (estimate −0.03; 95% CI −0.04 to −0.02; *p* < 0.001).

The same models were also calculated for the subset of patients with target height information. Target height SDS added significantly to the longitudinal variation of height SDS (model 3: estimate 0.52; 95% CI 0.35–0.69; *p* < 0.001) independently of all other associations identified in the total cohort, the effects of which remained significant in this subgroup.

## Discussion

This study provides an update on the growth performance and the major factors associated with statural growth in European children with advanced CKD. The cohort comprised a population of schoolchildren and adolescents with rather advanced CKD at study entry. Standardized height of the cohort averaged at −1.5 SDS at time of enrolment and decreased during prospective observation at a very slow pace, i.e., approximately 0.25 SDS per 5 years. The apparently largely stable growth performance of the overall cohort is the net result of a number of factors impeding and facilitating growth in the individual patient, which the large size of this cohort allowed to study by multivariable modeling. We found no associations of height at study entry and/or prospective growth rates with the type of underlying kidney disease. Previous studies suggested that the longer CKD exposure in patients with congenital disorders or disease-specific interference with growth mechanisms, such as more severe electrolyte and acid-base imbalance in tubulointerstitial disorders or metabolic nephropathies, may lead to more severe growth failure. While we cannot exclude that subtle differences in subsets of the main disease categories escaped detection, it appears more likely that tubular dysfunction was less relevant in this population with advanced CKD. However, we noticed significantly shorter statures both at study entry and during follow-up in children diagnosed with a defined syndrome. This was not unexpected since many syndromic disorders featuring renal abnormalities include growth failure as a phenotype. Our study highlights the need to account for the presence of syndromic disease when assessing growth in the pediatric CKD population.

Likewise, adjustment for the genetic target height, available in 80% of the patients, markedly improved the fit of the models. Per 1 SD unit of target height, the actual height SDS varied by 0.5 SD both at baseline and during follow-up. Hence, our findings support the consideration of the genetic growth potential as judged from parental heights, both in population studies and at patient level in the clinic.

Furthermore, two anthropometric indices were consistently associated with standardized height both at baseline and during follow-up: pubertal status and BMI SDS. The taller height observed in patients with clinical signs of puberty indicates an intact pubertal growth spurt. The apparent *gain* in standardized height may be due to the dyssynchrony of the pubertal growth spurt between children with CKD and the healthy reference population. Puberty tends to be delayed by 1–2 years in CKD, leading to substantial height gain at an age when growth has already ceased in healthy adolescents. As a result of this phase shift, height SDS tends to decrease in the late prepubertal period followed by an increase during puberty. The small but consistent negative association of BMIS SDS and height SDS both at baseline and during prospective observation might be surprising at first glance since an adequate nutritional status is a prerequisite for appropriate longitudinal growth in children with CKD. We speculate that in patients with BMI in the overweight and obese range intensified dietary efforts including enteral nutrition were applied during infancy to correct poor growth rates. Indeed, it has been demonstrated that aggressive enteral feeding in infants with CKD may result in excessive weight gain with rather limited effects on longitudinal growth (15, 16). It has also been shown that overweight acquired in infancy tends to persist in later childhood (17). Another contributing factor might be the association of obesity, short stature and CKD that is characteristic of some of the syndromic disorders represented in our cohort.

Renal function was consistently associated with height SDS, both at study entry and during follow-up. Patients with end stage kidney disease, in particular those who started dialysis during the observation period exhibited the poorest growth rates. This effect persisted after adjustment for markers of metabolic acidosis, hyperparathyroidism, anemia, and inflammation, suggesting that other factors associated with the uremic state are more important in inhibiting growth in end-stage kidney disease. Recent evidence suggests that better growth rates can be accomplished with hemodiafiltration as compared to standard hemodialysis (18, 19), providing renewed support to the old hypothesis that circulating inhibitors in the middle molecule range may accumulate in end-stage kidney disease (20, 21).

Recombinant GH is an efficacious therapy of uremic growth failure, which can improve final height with few side effects (22). Clinical practice recommendations regarding GH usage in children with CKD have been provided by expert committees (23, 24). In this study GH treatment, administered in 15% of patients for an average of three quarters of the observation time, was associated with a small but consistent positive effect on the course of height SDS. This observation reconfirms the role of recombinant GH as the only effective therapy of growth failure in children with CKD. The fact that 85% of children with a mean height below the third percentile did not receive GH documents the massive underutilization of this therapy in children with CKD. Reasons for low GH usage may include lacking approval and/or reimbursement issues, country policies, insufficient attention to growth failure by treating physicians, and families' reluctance to administer daily injections (25, 26). Particularly striking was the low use of GH in the two countries with the highest proportions of children with short stature, i.e., Turkey and the UK. While GH is not approved in Turkey, a very restrictive national policy focusing on nutritional management of growth is applied in the UK, where 42% of patients were found to be overweight and 39% stunted.

Even when taking into account all the factors discussed above, the addition of the country of residence to the multivariate models indicated additional country-specific factors impacting significantly on longitudinal growth. We can only speculate about the causes of this additional variation, which may include differences in the timing of diagnosis and/or referral to specialist care related to the organization of the national health care systems.

The strengths of this study include the long-term follow-up of a large representative contemporaneous multinational European patient cohort, the detailed patient-level information collected, and the use of novel reference datasets accounting for the secular trend of height and the substantial North-South gradient of height distribution across Europe. A limitation of our study is its confinement to children aged 6 years and older, which was due to the primary focus of the 4C Study on cardiovascular status assessment. Also, our analysis is limited to the assessment of growth on conservative treatment and dialysis since post-transplant growth was beyond the focus of this study.

In conclusion, we identified parental height and syndromic disease, pubertal status and BMI, end-stage renal disease and dialysis, GH treatment and the country of residence as key factors impacting on growth in children with CKD whereas renal anemia, metabolic acidosis and CKD-MBD did not appear to be of relevant influence.

## Data Availability

All datasets generated for this study can be provided upon request.

## Ethics Statement

This study was carried out in accordance with the Declaration of Helsinki and all EU and national recommendations for human studies with written informed consent from all subjects. All subjects gave written informed consent in accordance with the Declaration of Helsinki. The protocol was approved by the Ethics Committee of Heidelberg University and all local Institutional Review Boards.

## Author Contributions

FS, UQ, and AM designed the 4C Study. FS, RoB, and MK contributed the conception and design of the analysis presented here and wrote the first draft of this manuscript. RoB and MK performed the statistical analysis. AA, JB, RS, YB, SM, SC, DP, JH, FM, RaB, KA, OS, ArZ, and AlZ implemented the local study infrastructures, contributed substantial numbers of patients, provided intellectual input regarding the interpretation of the findings, and critically reviewed and revised the manuscript. All authors read and approved the submitted version.

### Conflict of Interest Statement

The authors declare that the research was conducted in the absence of any commercial or financial relationships that could be construed as a potential conflict of interest.
