# Supplementary material for: Determinants of Statural Growth in European Children With Chronic Kidney Disease: Findings From the Cardiovascular Comorbidity in Children With Chronic Kidney Disease (4C) Study
Source: Front Pediatr. 2019 Jul 5;7:278. doi: 10.3389/fped.2019.00278 (PMC6625460; doi:10.3389/fped.2019.00278)
Supplement: Supplementary file 1 [file Table_1.DOCX]

**Online Supplement**

**Table S-1**: Factors associated with height SDS at time of study enrolment in the total cohort.

|  | **Estimate** | **95% confidence interval** | **P value** |
| --- | --- | --- | --- |
| **Intercept** | -0.74 | -1.39; -0.09 | 0.026 |
| Age (years) | -0.06 | -0.11; -0.01 | 0.027 |
| Female sex | -0.16 | -0.41; 0.08 | 0.190 |
| Diagnosis |  |  |  |
| - CAKUT (reference) | 0 | - | - |
| - Glomerulopathies | 0.16 | -0.27; 0.59 | 0.457 |
| - Tubulointerstitial disorders | 0.08 | -0.25; 0.42 | 0.624 |
| - Post-AKI CKD | 0.03 | -0.52: 0.57 | 0.924 |
| - Other | 0.26 | -0.33; 0.85 | 0.391 |
| Defined syndrome | -0.34 | -0.68; -0.01 | 0.045 |
| Status: pubertal | 0.75 | 0.41; 1.09 | <0.001 |
| BMI SDS | -0.13 | -0.21; -0.04 | 0.003 |
| eGFR (per 10 ml/min/1.73 m^2^) | 0.18 | 0.10; 0.26 | <0.001 |
| Previous time on GH (years) | 0.05 | -0.05; 0.14 | 0.344 |
| Country of residence |  |  |  |
| - Germany (reference) | 0 | - | - |
| - Turkey | -1.27 | -1.61; -0.93 | <0.001 |
| - Serbia | -1.04 | 1.77; -0.31 | 0.005 |
| - Italy | -0.73 | -1.21; -0.24 | 0.003 |
| - France | -0.73 | -1.20; -0.25 | 0.003 |
| - UK | -0.59 | -1.15; -0.03 | 0.041 |
| - Austria | -0.44 | -1.14; 0.27 | 0.229 |
| - Poland | -0.28 | -0.82; -0.26 | 0.310 |
| - Other countries | -0.48 | -1.06; 0.11 | 0.114 |

**Table S-2**: Factors associated with height SDS during prospective observation period including time interactions.

|  |  | **Model 3.1 (AIC=4855.5)** |  |
| --- | --- | --- | --- |
|  | **Estimate** | **95% CI** | **P value** |
| **Intercept** | -0.77 | -1.37; -0.17 | 0.012 |
| Time (years) | -0.04 | -0.16; 0.07 | 0.450 |
| Age at enrolment (years) | 0.00 | -0.03; 0.04 | 0.841 |
| Female sex | -0.14 | -0.38; 0.11 | 0.277 |
| Syndromic disease | -0.39 | -0.73; -0.05 | 0.026 |
| Status: pubertal | 0.16 | 0.12; 0.20 | <0.001 |
| BMI SDS | -0.09 | -0.12; -0.07 | <0.001 |
| Cum. hospitalisation time (mo) | -0.07 | -0.16 ; 0.01 | 0.099 |
| Cum. time on GH (yrs) | 0.16 | 0.11; 0.21 | <0.001 |
| CKD stage |  |  |  |
| - 2 | 0.18 | -0.01; 0.37 | 0.066 |
| - 3a | 0.01 | 0.01; 0.20 | 0.049 |
| - 3b (reference) | 0.00 | - | - |
| - 4 | 0.03 | -0.03; 0.09 | 0.351 |
| - 5 | -0.03 | -0.12; 0.06 | 0.489 |
| - Dialysis | -0.02 | -0.14; 0.09 | 0.687 |
| Hemoglobin (g/dl) | 0.00 | -0.02; 0.02 | 0.975 |
| Serum albumin (g/L) | -0.00 | -0.01; 0.00 | 0.668 |
| Serum bicarbonate (mM) | 0.00 | -0.01; 0.01 | 0.822 |
| Serum phosphorus (mM) | -0.03 | -0.07; 0.02 | 0.252 |
| log (PTH (uM)) | -0.00 | -0.02; 0.02 | 0.925 |
| log (CRP (mg/L)) | 0.00 | -0.01; 0.02 | 0.545 |
| Country of residence |  |  |  |
| - Germany (reference) | 0.00 | - | - |
| - Turkey | -1.14 | -1.47; -0.80 | <0.001 |
| - Serbia | -0.87 | -1.60; -0.13 | 0.021 |
| - Italy | -0.77 | -1.26; -0.28 | 0.002 |
| - France | -0.59 | -1.06; -0.11 | 0.016 |
| - UK | -0.63 | -1.19; -0.06 | 0.031 |
| - Austria | -0.40 | -1.12; 0.32 | 0.279 |
| - Poland | -0.08 | -0.63; 0.46 | 0.767 |
| - Other countries | -0.48 | -1.07; 0.12 | 0.118 |
| Time interactions |  |  |  |
| - BMI SDS | -0.03 | -0.04; -0.02 | <0.001 |
| - CKD stage |  |  |  |
| - 2 | -0.05 | -0.13; 0.03 | 0.186 |
| - 3a | -0.04 | -0.08; 0.01 | 0.126 |
| - 3b (reference) | 0.00 | - | - |
| - 4 | -0.02 | -0.05; 0.00 | 0.091 |
| - 5 | -0.02 | -0.06; 0.01 | 0.172 |
| - Dialysis | -0.05 | -0.09; -0.01 | 0.015 |
| - Hemoglobin (g/dl) | -0.00 | -0.01; 0.00 | 0.517 |
| - Serum albumin (g/L) | 0.00 | -0.00; 0.00 | 0.543 |
| - Serum bicarbonate (mM) | -0.00 | -0.00; 0.00 | 0.646 |
| - Serum phosphorus (mM) | 0.00 | -0.01; 0.02 | 0.678 |
| - log(PTH (uM)) | 0.01 | -0.00; 0.02 | 0.120 |
| - log(CRP (mg/L)) | -0.01 | -0.01; 0.00 | 0.083 |
